# Caffeine discontinuation improves acute migraine treatment: a prospective clinic-based study

**DOI:** 10.1186/s10194-016-0662-5

**Published:** 2016-08-05

**Authors:** Mi Ji Lee, Hyun Ah Choi, Hanna Choi, Chin-Sang Chung

**Affiliations:** 1 Department of Neurology, Neuroscience Center, Samsung Medical Center, Sungkyunkwan University School of Medicine, 81 Irwon-Ro, Gangnam-Gu, Seoul 135-710 South Korea; 2 Department of Neurology, Eulji University Hospital, Eulji University School of Medicine, Daejeon, South Korea

**Keywords:** Migraine, Caffeine, Acute treatment

## Abstract

**Background:**

Caffeine has both excitatory and vasoconstrictive effects on central nervous system. Caffeine use might be associated with development and chronification of migraine. We aimed to evaluate the effect of caffeine cessation on the acute treatment of migraine.

**Methods:**

We prospectively recruited migraine patients who consumed caffeine drinks daily and instructed them to discontinue their caffeine intake. Triptans were prescribed for acute treatment. Patients were followed up after at least two weeks after screening and evaluated the efficacy of acute treatment with the migraine assessment of current therapy (Migraine-ACT) questionnaire. Excellent efficacy was defined as Migraine-ACT score of 4. Chronic migraine, body mass index, allodynia, depression, anxiety, antiemetic use, and use of prophylactic medication were included in the multivariate analysis if the univariate *p* < 0.2.

**Findings:**

Among 108 patients included, 36 completely discontinued their caffeine intake (abstinence group). The efficacy of acute treatment was assessed at median 34.5 days (interquartile range, 28–89) after the screening. Twenty-six patients (72.2 %) in the abstinence group and 29 (40.3 %) in the non-abstinence group reported an excellent efficacy (*p* = 0.002). The abstinence group also showed a trend toward greater reduction of headache impact test-6 (HIT-6) scores (*p* = 0.085). Caffeine abstinence was independently associated with an excellent efficacy of acute treatment (multivariate odds ratio, 3.2; 95 % confidence interval, 1.2–8.4; *p* = 0.018) after controlling for covariates.

**Conclusions:**

Caffeine abstinence is associated with better efficacy of acute migraine treatment. Our uncontrolled study results encourage a further confirmatory study on this issue.

## Introduction

Caffeine, the most popular psychostimulant drug in the world [1], may act as a double-edged sword in migraine patients. Caffeine has been used for the adjuvant treatment of acute migraine attacks. High caffeine consumption is associated with the development and chronification of migraine, although the association is not strong [2–4].

There are conflicting reports regarding the action of caffeine on pain. Previous studies have shown a small but significant additive effect of caffeine for the control of headache and non-headache pain [5, 6]. However, preclinical studies showed that caffeine has an intrinsic antinociception at extremely high doses (25–100 mg/kg) but also can inhibit antinociception at lower doses [7]. If caffeine has intrinsic analgesic effect, it is also possible that chronic caffeine use may induce a medication-overuse state in migraineurs. In contrast, if caffeine has anti-nociceptice effect, it should affect on the outcome of acute headache treatment. Both assumptions may warrant a prospective study regarding impact of caffeine cessation on acute migraine treatment. However, there has been no prospective study addressing the impact of caffeine cessation in migraineurs.

Through competitive inhibition of adenosine receptor, caffeine results in vasoconstriction on cerebral blood vessels [8, 9]. Acute withdrawal of caffeine is therefore related with rebound cerebral vasodilatation, which may be a cause of caffeine withdrawal headache. However, the rebound increase of cerebral blood flow can be restored after 2 weeks of caffeine abstinence [9]. In the present study, we aimed to evaluate the effect of caffeine cessation for at least 2 weeks on the outcome of acute migraine treatment in a prospective setting.

## Methods

### Patients

We prospectively recruited consecutive first-visit patients who visited the Samsung Medical Center headache clinic from Mar 2015 and Sep 2015. Patients who (1) were diagnosed with migraine, and (2) consumed any kind of caffeinated drink on a daily basis were considered eligible for the study. Patients were excluded if they (1) had medication overuse headache at the time of screening, (2) were diagnosed with probable migraine, (3) were contraindicated to oral triptans (serotonin 1B/1D receptor agonist), (4) had active psychiatric illnesses, or (5) refused to participate in the scheduled follow-up evaluation. Among them, patients who took acute medications at least three times before the follow-up study were finally analyzed. The diagnosis of migraine, probable migraine, and medication overuse headache was based on the international classification of headache disorders, 3^rd^ edition beta version (ICHD-3 beta) [10]. The Samsung Medical Center Institutional Review Board approved this study.

### Evaluations

The daily dose and sources of caffeine were assessed by both a questionnaire and interview. Sources of caffeine included brewed or drip coffee, instant coffee, caffeinated tea, cola, and energy drink, with estimated doses of 136 mg, 96 mg, 40 mg, 36 mg, and 80 mg per 8-oz serving, respectively [11].

The baseline assessment included a structured headache questionnaire, which evaluated the headache characteristics, headache frequencies, vascular risk factors, smoking, and alcohol intake. The headache-related quality of life was assessed using the Korean version of the headache impact test-6 (HIT-6) in a previously validated form [12]. The Hospital Anxiety and Depression Scale (HADS) was used for screening comorbid depression and anxiety disorders. Recent depression was defined as a HADS-depression (HADS-D) score of ≥8, and recent anxiety as a HADS-anxiety (HADS-A) score of ≥8. Allodynia was assessed with the allodynia symptom checklist-12 (ASC-12) [13]. Patients were classified as allodynic when they had ASC-12 scores of ≥3 [13].

### Intervention

All the patients were instructed by the investigators (C.-S.C., M.J.L, H.C.) to abruptly discontinue caffeine intake. Pharmacological treatments were not controlled in this study and prescribed at the physician’s discretion. Triptan agents were used for the acute abortive medication. Different triptans were individually prescribed per the patient’s headache characteristics, such as headache duration, time to peak intensity, and recurrence pattern [14, 15]. Antiemetics were given to patients if they reported prominent nausea or vomiting during their typical headache attack. Simple analgesics or non-steroidal anti-inflammatory drugs (NSAIDs) were not prescribed in this study. All the patients were instructed to take their acute medication before the headache became severe. Caffeinated medications were prohibited. Preventive medications were prescribed at the physician’s discretion. Patients were instructed to record their daily consumption of caffeinated drinks, acute abortive medication use, and the efficacy of the medication in a daily headache diary.

### Follow-up

To evaluate impacts of caffeine cessation after resolution of rebound cerebral vasodilation and consequent caffeine-withdrawal headache, patients were followed up at least 2 weeks after the screening [9, 10]. Ideally, the first follow-up visit was planned at 4 ± 2 weeks. Acute medications were not changed during the observation period. At the follow-up visit, the caffeine intake was assessed based on the headache diary and an interview by the physicians. Caffeine abstinence was defined as the complete cessation of any caffeinated agents. If caffeine had not been discontinued (“non-abstinence”), patients were sub-classified as “reduction” and “non-reduction” based on the average daily dose of caffeinated drinks.

Patients were instructed to complete the structured follow-up questionnaire, which included questions on the monthly frequency of headaches, the number of acute abortive medications taken per month, and the HIT-6 form [12]. Patients completed the migraine assessment of current therapy (migraine-ACT) questionnaire [16] when the cumulative use of acute treatment was more than three times. If a patient took the acute medication less than three times, he or she was followed up again to evaluate efficacy of at least three trials of acute treatment. The migraine-ACT questionnaire is a validated method to assess the consistency (item 1), global assessment of relief (item 2), impact (item 3), and emotional response (item 4) associated with the acute migraine medication [16].

### Statistical analysis

The chi-square and Fisher’s exact tests were performed to compare the categorical variables between the groups. Student’s *t*-test was used for the continuous variables. Excellent efficacy of acute treatment was defined as an ACT score of 4. Daily dose of caffeine consumption was categorized as ≥200, <200, and 0 mg/day and tested by using a linear-by-linear association tests in relation to ACT subscores. HIT-6 scores were compared only in patients with a follow-up interval of more than 1 month.

Univariate and multivariate logistic regression analysis was performed to determine the independent effect of caffeine cessation on the rate of excellent efficacy of acute treatment. Confounders, including chronic migraine, body mass index, allodynia, depression, anxiety, antiemetic use, different triptans, and use of prophylactic medication, were tested by using a forward stepwise multivariate logistic regression model. Daily dose of caffeine intake at the follow-up was also tested in the aforementioned univariate and multivariate logistic models.

To test if there is an impact of higher (>200 mg/day) baseline caffeine consumption on the caffeine cessation and efficacy of acute headache treatment, an interaction analysis was performed with multivariate logistic regression analysis.

Statistical analyses were performed using the commercially available SPSS software version 18.0 (IBM, North Castle, NY, USA). A *p* value <0.05 was considered statistically significant.

## Findings

Among the 113 eligible patients, we included 108 who took their acute medication at least 3 times before the follow-up visit. At the follow-up, 36 (33.3 %) patients had discontinued the consumption of caffeine (“abstinence group”), while 72 (66.7 %) had not (“non-abstinence group”).

The patients’ demographics are shown in Table 1. The abstinence group had a longer history of migraine (*p* = 0.010) and higher baseline HIT-6 scores (*p* = 0.018) than the non-abstinence group. Both groups had frequent migraine attacks with median headache days of 9 per month. Otherwise, there were no significant differences in the demographics, comorbidities, and headache characteristics between the two groups.

**Table 1:** Baseline characteristics of patients

	Abstinence group (*N* = 36)	Non-abstinence group (*N* = 72)	*P*
*Baseline*			
Age, y	41.0 (35.3 – 53.8)	44.0 (37.0 – 55.0)	0.379
Females	30 (83.3 %)	54 (75.0 %)	0.326
BMI, kg/m^2^	21.3 (19.5 – 24.4)	22.8 (20.5 – 24.6)	0.141
Disease duration, years	10.0 (4.8 – 21.0)	4.5 (2.0 – 10.0)	0.010
Type of primary headache			0.637
Migraine without aura	26 (72.2 %)	55 (76.4 %)	
Migraine with aura	10 (27.8 %)	17 (23.6 %)	
Chronic migraine	12 (33.3 %)	21 (29.2 %)	0.658
Allodynia (ASC ≥3)	9 (25.0 %)	15 (22.1 %)	0.735
Headache days (/month)	9.0 (3.0 – 20.0)	9.0 (4.0 – 25.0)	0.546
Severity of headache (numeric rating scale)	7.0 (6.1 - 8.5)	7.0 (6.0 - 8.3)	0.967
HIT-6 score	63.0 (60.0 – 67.5)	60.0 (56.0 – 65.0)	0.018
Pretreatment HADS score	14.0 (11.5 – 17.0)	13.0 (10.0 – 20.0)	0.888
Psychiatric comorbidity			
Depression	9 (36.0 %)	20 (46.5 %)	0.398
Anxiety disorder	12 (46.2 %)	16 (37.2 %)	0.463
Estimated dose of caffeinated drinks (mg/day)	192.0 (96.0 – 192.0)	192.0 (96.0 – 288.0)	0.285
Prophylactic medication	25 (34.7 %)	17 (47.2 %)	0.209
Topiramate	5 (13.9 %)	5 (6.9 %)	0.241
Beta-blockers	12 (33.3 %)	15 (20.8 %)	0.157
Calcium channel blockers	14 (38.9 %)	11 (15.3 %)	0.006
Antidepressants	11 (30.6 %)	17 (23.6 %)	0.438
Antiemetics combination	25 (69.4 %)	49 (68.1 %)	0.884
*Follow-up*			
Follow-up interval (days)	31.0 (28.0 – 84.3)	35.0 (20.3 – 87.5)	0.894
Estimated dose of caffeinated drinks during follow-up (mg/day)	0.0 (0.0 – 0.0)	96.0 (96.0 – 168.0)	<0.001
<200		54 (75.0 %)	
≥200		18 (25.0 %)	
Headache days in the last month	8.5 (4.0 – 15.0)	7.0 (3.0 – 12.0)	0.330
Severity of headache (numeric rating scale)	5.5 (4.0 - 7.0)	6.0 (4.8 - 7.0)	0.117
Acute medication use in the last month	5.0 (4.0 – 10.0)	5.0 (3.5 – 7.5)	0.369
HIT-6 score ^a^	59.0 (52.0 – 65.0)	60.0 (55.0 – 62.0)	0.547
HIT-6 improvement ^a^	5.0 (1.0 – 10.0)	2.0 (-2.3 – 7.0)	0.085
ACT score	4.0 (2.3 – 4.0)	3.0 (1.0 – 4.0)	0.002
ACT = 4	26 (72.2 %)	29 (40.3 %)	0.002

Values are presented as N (%) or median (interquartile range)

Abbreviations: *SD* standard deviation, *BMI* body mass index, *HADS* headache depression and anxiety scale, *HIT-6* headache impact test-6, *ACT* assessment of current treatment

^a^ HIT-6 scores were compared only in 79 patients with follow-up of >1 month

**Fig. 1:**
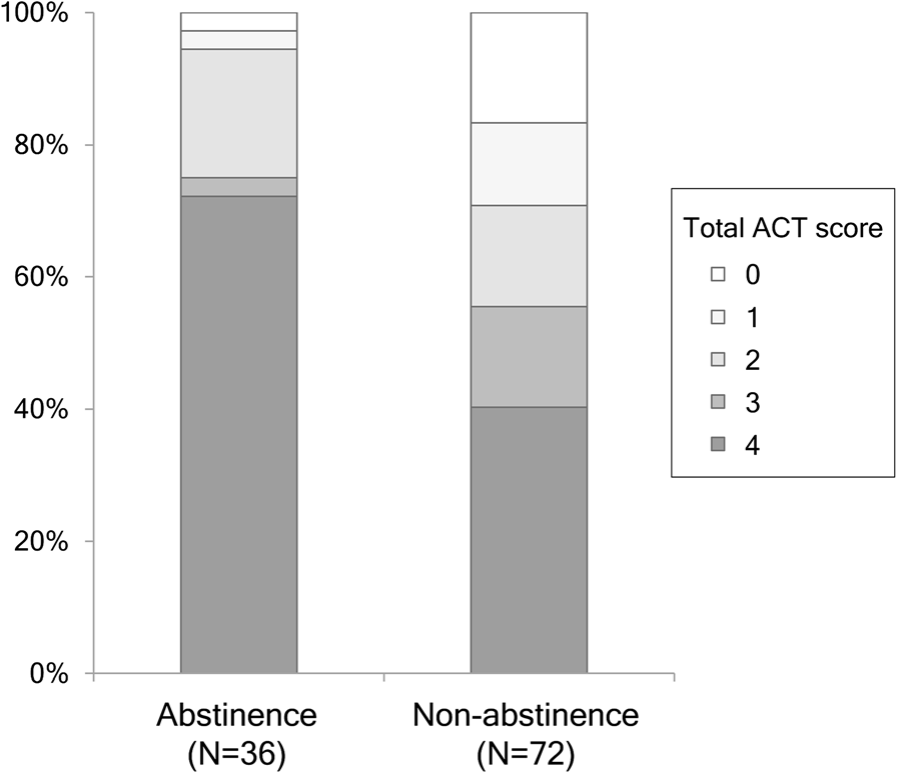
The distribution of total scores in the assessment of current treatment (Migraine-ACT)

Median interval from the first visit to second visit was 31 (interquartile range [IQR] 21 – 37) days. Twenty-three patients who took their medication less than three times were followed up again. Finally, all patients completed the efficacy of acute treatment at median 34.5 (IQR 28 – 89) days after the screening. Twenty-six patients (72.2 %) in the abstinence group reported an excellent efficacy, which is significantly higher than in the non-abstinence group (40.3 %, *p* = 0.002; Table 1). The distribution of the ACT scores is shown in Fig. 1. Among 79 patients with more than 1-month follow-up, the cessation group (*n* = 29) also showed a trend toward greater reduction of HIT-6 scores than non-abstinence group (*n* = 50; *p* = 0.085, Table 1). There were no differences in headache intensity and monthly headache days between the abstinence and non-abstinence groups.

**Fig. 2:**
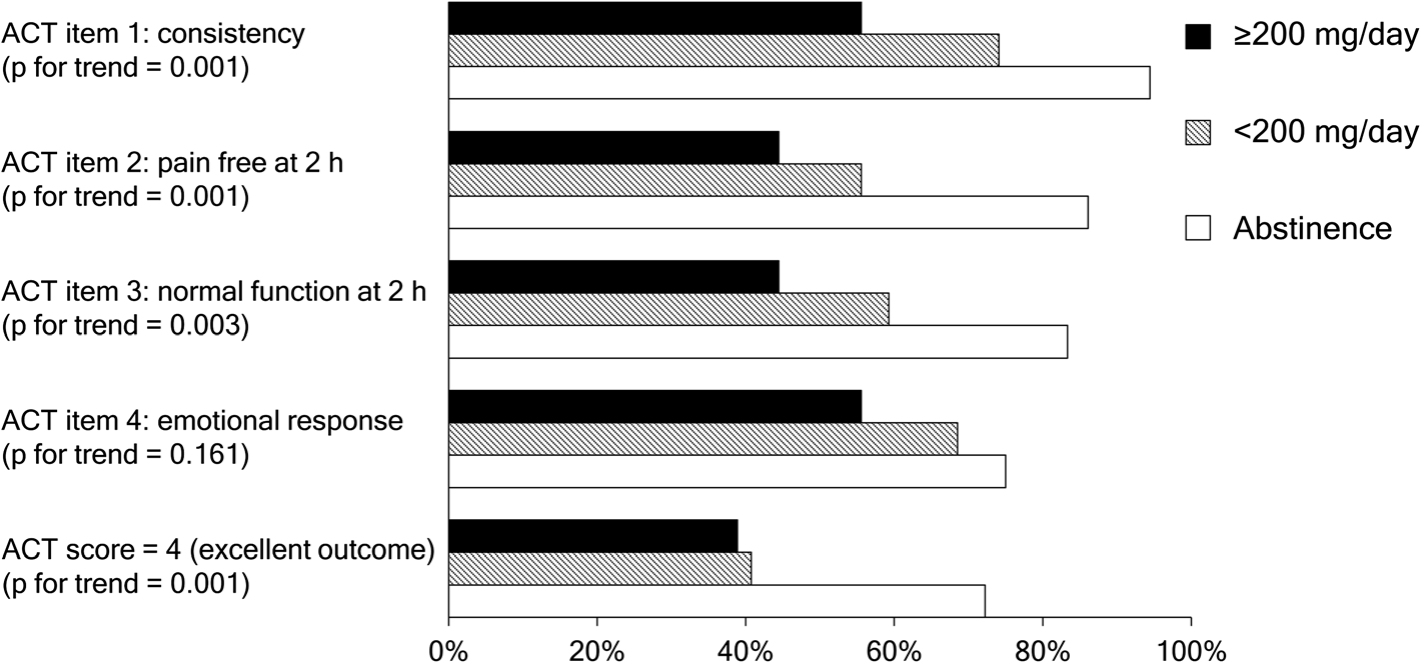
Proportion of positive responses for each item of the Migraine-ACT questionnaire, according to the subgroups of high (≥200 mg/day), low (<200 mg/day), and no consumption of caffeinated agents. *The Migraine-ACT questionnaire is comprised of: item 1 (Consistency of response), item 2 (Disappearance of pain within 2 h), item 3 (Ability to function normally within 2 h), and item 4 (Feeling comfortable enough to be able to plan daily activities)

A dose-dependent relationship was present between the daily dose of caffeinated agents and the components of the ACT questionnaire (Fig. 2). A significant trend was found between daily caffeine dose and ACT subscores of consistency, 2-h pain free response, functioning, and the total score (p for trend = 0.001, 0.001, 0.003, and 0.001), but not in the emotional assessment (p for trend = 0.161).

Univariate analysis revealed significant variables associated with the excellent efficacy of acute therapy. Caffeine abstinence (univariate OR 3.9, 95 % CI 1.6–9.2, *p* = 0.002) was associated with excellent efficacy. The daily consumption of caffeinated drinks showed a negative dose-dependent association with the excellent efficacy of acute treatment (OR 0.5, 95 % CI 0.3–0.9 per an increase of 100 mg caffeine/day, *p* = 0.015). In the multivariate analysis, caffeine abstinence was independently associated with an excellent efficacy of acute treatment (multivariate OR 3.2, 95 % CI 1.2–8.4, *p* = 0.018) after controlling for significant covariates. There was no interaction of baseline caffeine consumption (≥200 vs <200 mg/day) and caffeine cessation on the efficacy of acute headache treatment (p for interaction = 0.814)

## Discussion

The main finding of our study is that the complete cessation of daily caffeine intake is independently associated with the excellent efficacy of acute treatment of migraine. A negative dose-dependent relationship was found between the daily caffeine intake and the efficacy of the acute migraine medication in multiple aspects.

In this observational study, we demonstrated a beneficial effect of caffeine cessation on the acute treatment of migraine. In the brain, caffeine result in an increased release of excitatory neurotransmitters through competitive inhibition of adenosine A1 receptor and vasoconstriction via A2 receptor antagonism on cerebral blood vessels [8]. Chronic caffeine intake may lead to the up-regulation of adenosine receptors and compensatory elevation in the plasma concentrations of adenosine, which is a potent vasodilator that precipitates migraine headaches [17, 18]. Therefore, vasoconstrictive effects of triptan may be negatively affected by daily caffeine intake. Although caffeine cessation might be beneficial in migraine treatment, it can be complicated by caffeine-withdrawal headache [19]. Rebound cerebral vasodilation, which is a mechanism of caffeine-withdrawal headache, can be normalized after 2 weeks of caffeine abstinence [9]. We therefore used at least 2-week discontinuation of caffeine in this study.

To date, there is no consensus recommendation how clinicians should explain about caffeine intake to migraine patients. We classified the baseline and follow-up caffeine consumption with a cutoff of 200 mg/day, which is associated with acute analgesic effect and caffeine-withdrawal headache [10, 18]. Our data showed that the baseline dose of caffeine did not affect the impact of caffeine cessation. That is, high-dose caffeine consumers as well as low- to moderate-dose consumers can benefit from the abrupt caffeine discontinuation. However, the dose of current caffeine intake was associated with different aspects of ACT scores in the present study. The outcome was even better after complete discontinuation than low-dose caffeine consumption. Taken together, our study results might indicate that migraine patients may benefit from complete abstinence of caffeine, similarly to the abrupt discontinuation of acute analgesics for detoxification in patients with medication overuse headache [20, 21].

Our study was not without limitations. First, we did not use the same triptan agents to all patients. Different triptan agents may have different efficacy. However, we adjusted the possible influence of different agents by using the multivariate analysis. Second, it is possible that the caffeine consumption was influenced by the efficacy of the acute medication. However, the most common reasons for non-cessation were a desire to stay awake, rather than as a rescue for acute headache attack. Third, caffeine cessation may represent a good compliance and doctor-patient relationship. However, in our quantitative analysis, doses of daily caffeine intake were related with functional but not with emotional assessment of acute treatment efficacy. Fourth, we did not prospectively collect data regarding caffeine-withdrawal headache. The risk of headache exacerbation could not be determined in our study.

In conclusion, our uncontrolled study suggest that caffeine cessation might be beneficial for acute migraine treatment. Further confirmatory studies may be necessary to prove the causal relationships.

## References

[1] Fulgoni VL, Keast DR, Lieberman HR (2015). Trends in intake and sources of caffeine in the diets of US adults: 2001-2010. Am J Clin Nutr.

[2] Hering-Hanit R, Gadoth N (2003). Caffeine-induced headache in children and adolescents. Cephalalgia Int J Headache.

[3] Scher AI, Stewart WF, Lipton RB (2004). Caffeine as a risk factor for chronic daily headache: a population-based study. Neurology.

[4] Hagen K, Thoresen K, Stovner LJ, Zwart JA (2009). High dietary caffeine consumption is associated with a modest increase in headache prevalence: results from the Head-HUNT Study. J Headache Pain.

[5] Derry CJ, Derry S, Moore RA (2014). Caffeine as an analgesic adjuvant for acute pain in adults. Cochrane Database Syst Rev.

[6] Ward N, Whitney C, Avery D, Dunner D (1991). The analgesic effects of caffeine in headache. Pain.

[7] Sawynok J (2011). Caffeine and pain. Pain.

[8] Fredholm BB, Battig K, Holmen J, Nehlig A, Zvartau EE (1999). Actions of caffeine in the brain with special reference to factors that contribute to its widespread use. Pharmacol Rev.

[9] Sigmon SC, Herning RI, Better W, Cadet JL, Griffiths RR (2009). Caffeine withdrawal, acute effects, tolerance, and absence of net beneficial effects of chronic administration: cerebral blood flow velocity, quantitative EEG, and subjective effects. Psychopharmacology.

[10] Headache Classification Committee of the International Headache S (2013). The International Classification of Headache Disorders, 3rd edition (beta version). Cephalalgia Int J Headache.

[11] Barone JJ, Roberts HR (1996). Caffeine consumption. Food Chem Toxicol Int J Published British Ind Biol Res Assoc.

[12] Chu MK, Im HJ, Ju YS, Yu KH, Ma HI, Kim YJ, Kim J, Lee BC (2009). Validity and reliability assessment of Korean headache impact test-6 (HIT-6). J Korean Neurol Assoc.

[13] Lipton RB, Bigal ME, Ashina S, Burstein R, Silberstein S, Reed ML, Serrano D, Stewart WF (2008). Cutaneous allodynia in the migraine population. Ann Neurol.

[14] Goadsby PJ, Sprenger T (2010). Current practice and future directions in the prevention and acute management of migraine. Lancet Neurol.

[15] Gladstone JP, Dodick DW (2004). Acute Migraine: Which Triptan?. Pract Neurol.

[16] Dowson AJ, Tepper SJ, Baos V, Baudet F, D’Amico D, Kilminster S (2004). Identifying patients who require a change in their current acute migraine treatment: the Migraine Assessment of Current Therapy (Migraine-ACT) questionnaire. Curr Med Res Opin.

[17] Johansson B, Georgiev V, Lindstrom K, Fredholm BB (1997). A1 and A2A adenosine receptors and A1 mRNA in mouse brain: effect of long-term caffeine treatment. Brain Res.

[18] Shapiro RE (2007). Caffeine and headaches. Neurol Sci Off J Italian Neurol Soc Italian Soc Clin Neurophysiol.

[19] Silverman K, Evans SM, Strain EC, Griffiths RR (1992). Withdrawal syndrome after the double-blind cessation of caffeine consumption. N Engl J Med.

[20] Chiang CC, Schwedt TJ, Wang SJ, Dodick DW (2016). Treatment of medication-overuse headache: A systematic review. Cephalalgia Int J Headache.

[21] Bendtsen L, Munksgaard S, Tassorelli C, Nappi G, Katsarava Z, Lainez M, Leston J, Fadic R, Spadafora S, Stoppini A, Jensen R, Consortium C (2014). Disability, anxiety and depression associated with medication-overuse headache can be considerably reduced by detoxification and prophylactic treatment. Results from a multicentre, multinational study (COMOESTAS project). Cephalalgia Int J Headache.

